# Assessing Laryngectomy Patient Education on YouTube: Investigating Quality and Reliability

**DOI:** 10.1002/oto2.113

**Published:** 2024-01-31

**Authors:** Matthew E. Lin, Oluwatobiloba Ayo‐Ajibola, Carlos X. Castellanos, Jonathan West, Neil Luu, Ian Kim, Niels C. Kokot

**Affiliations:** ^1^ Keck School of Medicine of the University of Southern California Los Angeles California USA; ^2^ Caruso Department of Otolaryngology–Head & Neck Surgery Keck School of Medicine of the University of Southern California Los Angeles California USA; ^3^ Departments of Pediatrics and Medicine Stanford University Stanford California USA; ^4^ Department of Kinesiology Pennsylvania State University University Park Pennsylvania USA

**Keywords:** head and neck cancer, laryngeal cancer, laryngectomy, quality, reliability, social media, YouTube

## Abstract

**Objective:**

This study aimed to characterize the quality of laryngectomy‐related patient education on YouTube and understand factors impacting video content quality.

**Study Design:**

Cross‐sectional cohort analysis.

**Setting:**

Laryngectomy‐related videos on YouTube.

**Methods:**

YouTube was anonymously queried for various laryngectomy procedure search terms. Video quality was evaluated using the validated DISCERN instrument which assesses treatment‐related information quality. Descriptive statistics were used to characterize our cohort. Univariate and multivariable linear regression were used to assess factors associated with increased DISCERN score. Significance was set at *P* < .05.

**Results:**

Our 78‐video cohort exhibited moderate levels of engagement, averaging 13,028.40 views (SD = 24,246.93), 69.79 likes (SD = 163.75), and 5.27 comments (SD = 18.81). Videos were most frequently uploaded to accounts belonging to physicians (43.59%) or health care groups (41.03%) and showcased operations (52.56%) or physician‐led education (20.51%). Otolaryngologists were featured in most videos (85.90%), and most videos originated outside the United States (67.95%). Laryngectomy videos demonstrated poor reliability (mean = 2.35, SD = 0.77), quality of treatment information (mean = 1.92, SD = 0.86), and overall video quality (mean = 1.97, SD = 1.12). In multivariable linear regression, operative videos were associated with lower video quality relative to nonoperative videos (*β* = −1.63, 95% confidence interval [CI] = [−2.03 to −1.24], *P* < .001); the opposite was true for videos from accounts with higher subscriber counts (*β* = 0.02, 95% CI = [0.01‐0.03], *P* = .005).

**Conclusion:**

The quality and quantity of YouTube's laryngectomy educational content is limited. There is an acute need to increase the quantity and quality of online laryngectomy‐related content to better support patients and caregivers as they cope with their diagnosis, prepare for, and recover from surgery.

Head and neck cancer (HNC) is a significant health concern in the United States, comprising 3% of all malignancies.^1^, ^2^ Laryngeal cancer is a subtype of HNC that primarily affects older, male patients, and accounts for around 12,460 new cases and 3820 deaths annually in the United States.^3^, ^4^ Laryngeal cancer is often treated with a combination of chemotherapy, radiation therapy, and surgical intervention through partial or total laryngectomy.^5^ This procedure imposes an immediate and significant physical and psychological toll on the patient's quality of life as it removes their ability to speak, at least in the short term, and fundamentally alters the way in which the patient interacts with the world around them.^6^, ^7^ As such, effective patient education is key to helping individuals understand the procedure, potential outcomes after surgery, and quality of life after laryngectomy.^8^

Historically, patient education has been led by physicians during clinic visits and supplemented by anecdotes from friends, family, and caregivers. However, barriers to physician visits such as long wait times and shorter visits can impede patients' access to information from their providers.^9^, ^10^ In particular, Langford et al. found shorter visit lengths were associated with lower perceived quality of patient‐provider communication and increased odds of seeking other means of medical education.^11^ The Internet's ubiquitous nature has presented patients with a growing number of sources of medical information and now frequently eclipses physicians as the initial source of medical information.^12^ YouTube—a free online video‐sharing platform used by over 80% of US adults^13^—can be a helpful resource for patients hoping to understand the risks, events, and quality of life after surgery, especially when the video content is sourced from medical professionals and academic institutions.^14^

Despite widespread prior investigations into the quality and credibility of YouTube videos for patient decision‐making and health care professional education, there is a lack of research applying this framework to laryngectomy content.^15^, ^16^, ^17^ Given the potential benefits and drawbacks of YouTube to educate patients, it is critical to understand its effectiveness in accurately educating patients seeking treatment information about laryngectomy. Thus, we aim to assess the quality and reliability of laryngectomy content on YouTube to understand factors impacting the quality of content available to patients. We hope to identify areas of improvement and guide the development of effective video educational resources for laryngeal cancer patients online and hypothesize content from health care organizations and physicians would rate significantly higher on the DISCERN scale.

## Methods

This study was exempt from review by the Institutional Review Board of the University of Southern California due to lack of patient involvement or personal identifying health information. This study involves a cross‐sectional cohort analysis of YouTube videos related to laryngectomy. All videos were anonymously queried in November 2022 on an incognito web browser to avoid account‐specific search biases. The following search terms were used: laryngectomy, total laryngectomy, partial laryngectomy, transoral robotic laryngectomy, salvage laryngectomy, stapler laryngectomy, conservation laryngectomy, vertical partial laryngectomy, supracricoid partial laryngectomy with cricohyoidoepiglottopexy, supracricoid partial laryngectomy with cricohyoidopexy, supraglottic laryngectomy, laryngopharyngectomy, total laryngopharyngectomy, and partial laryngopharyngectomy.

Video metrics were collected, including video length, age of video in months, age of posting account in months, and the number of likes, views, comments, and account subscribers. Videos were also classified by type of user account (physician, health care group, academic institution, device company, academic society, entertainment), type of video category (patient experience, education from patient, education from physician, operation, entertainment), country of origin (United States and outside United States), and physician specialty (otolaryngology vs not) when relevant. Health care groups were defined as clinics, physician groups, and other collections of health care professionals who provide clinical care. Academic societies were defined as professional membership organizations, and entertainment accounts were defined as accounts that publish noneducational content. Educational videos were defined as those created for informative purposes and were either patient or physician‐driven. Patient experience videos were defined as those focused on patients—emphasizing the process of diagnosis and expectations regarding perioperative management. Operational videos showcased laryngectomies intraoperatively. Entertainment videos were defined as those whose primary intention was viewer enjoyment rather than education.

The DISCERN scale—a 16‐question, 5‐point validated scale assessing treatment‐related information quality where higher scores indicate higher quality content—was used to assess the informational quality of laryngectomy‐related videos on YouTube.^18^ This scale has been extensively used to evaluate the quality of other treatment‐related online videos and splits its 15 questions into 3 categories: reliability, quality, and overall rating. In this score, reliability refers to the trustworthiness of the content, whereas quality refers to the accuracy and specificity of information provided. Scores 4.5 and higher are considered excellent, those from 4.2 to 4.4 are considered very good, 3.4 to 4.1 good, 2.6 to 3.3 average, 1.9 to 2.5 poor, and less than 1.8 very poor. Three reviewers analyzed a subset of videos together based on DISCERN handbook guidelines to limit inter‐rater variability.

Data analysis was performed in R 2022.07.2 (R Group for Statistical Computing) and Microsoft Excel (version 16.71, Microsoft Software). Descriptive statistics were used to characterize our cohort; frequencies and percentages were used for categorical variables and means and standard deviations were used for continuous variables. Two‐sided *t* test and Pearson's *χ* ^2^ test were used to assess for significant differences between numeric and factorial variables, respectively. Univariate and multivariable linear regression was used to assess for factors associated with increased DISCERN score. Given the limited sample size of videos available, the forward stepwise variable selection method was used to produce a concise final model that maintains statistical power; in this method, variables are added one at a time, variables which change parameter coefficients by over 15% are assessed for confounding, and all confounding variables and the vast majority of nonsignificant variables are removed from the final model.^19^

Linear regression outputs were reported as *β* coefficients with 95% confidence interval (CI). For DISCERN score, all *β*s were reported per 1 unit increase in score of each predictor variable. For example, a *β* of 0.5 indicates that if a predictor score increased by 1 unit, the DISCERN score increased by 0.5. Binary variables such as video type were coded as 0 (absence) or 1 (presence). Thus, a 1 unit increase of each binary variable represented the presence of the variable. For all statistical tests, the level of significance was set at *P* < .05.

## Results

### Video Demographics

Table 1 delineates characteristics and metrics of the 78 YouTube videos related to laryngectomy included in this study. On average, videos had 13,028.40 views (SD = 29.246.93), 69.79 likes (SD = 163.75), and 5.27 comments (SD = 18.81). Average video length was 14.90 minutes (SD = 21.29, range = 1‐143 minutes). Videos were posted on average 58.50 months (SD = 50.21) ago by accounts with an average age of 119.72 months (SD = 53.25). Most videos were posted by accounts originating from outside of the United States (n = 53, 68.95%) with average subscriber count of 54,232.60 (SD = 184,612.63). The most common user account types were physicians (n = 34, 43.59%) and health care groups (n = 32, 41.03%). The most common video types were operative (n = 41, 52.56%), physician‐led education (n = 16, 20.51%), and patient experiences (n = 11, 14.10%). Most videos featured otolaryngologists (n = 67, 85.90%).

**Table 1:** Video Metrics and Characteristics

Characteristic	n (%) n = 78
Video and account metrics (mean, SD)	
Length, min	14.90 (21.29)
Video age, mo	58.50 (50.21)
Likes (#)	69.79 (163.75)
Views (#)	13,028.40 (29,246.93)
Comments (#)	5.27 (18.81)
Account subscribers (#)	54,232.60 (184,612.63)
Account age, mo	119.72 (53.25)
Type of user account	
Physician	34 (43.59)
Health care group	32 (41.03)
Academic institution	2 (2.56)
Device company	1 (1.28)
Academic society	7 (8.97)
Entertainment	2 (2.56)
Type of video	
Operation	41 (52.56)
Education from physician	16 (20.51)
Patient experience	11 (14.10)
Education from patient	8 (10.26)
Entertainment	2 (2.56)
Otolaryngologist featured	67 (85.90)
Geographic origin	
United States	25 (32.05)
Outside United States	53 (67.95)

### DISCERN Scores

**Figure 1:**
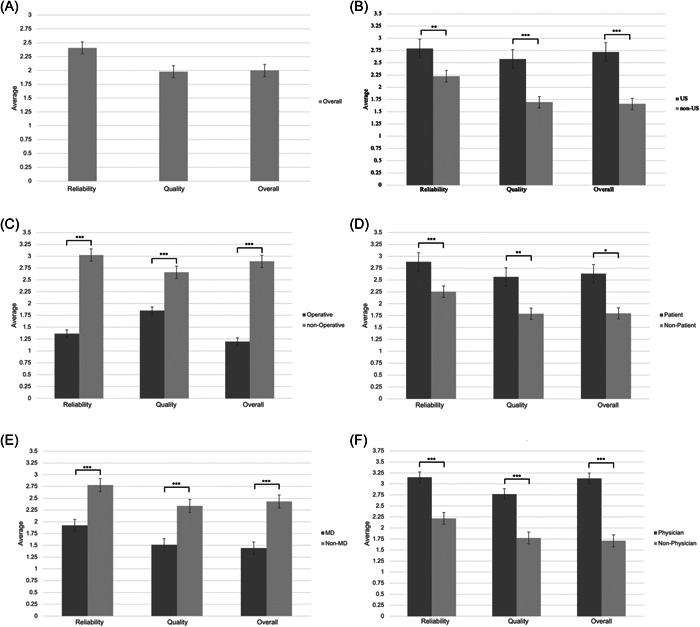
Reliability, quality, and overall DISCERN scores. (A) All videos. (B) United States versus non‐United States. (C) Operative versus nonoperative. (D) Patient versus nonpatient‐focused. (E) Posted to MD versus non‐MD account. (F) Featuring physicians versus not.

The validated DISCERN scale was applied to assess video content quality and reliability, as illustrated in Figure 1.

Videos created by patients exhibited significantly higher overall DISCERN scores relative to videos not created by patients (2.63 vs 1.80, *P* = .015). YouTube media featuring operative content scored significantly lower compared to nonoperative (1.20 vs 2.89, *P* < .001). Videos featuring physicians exhibited significantly greater overall DISCERN scores relative to nonphysician featuring videos (3.125 vs 1.71, *P* < .001); videos posted to non‐MD health care accounts exhibited greater video quality than those featured on physician‐owned accounts (1.44 vs 2.43, *P* < .001). Videos originating from the United States also had significantly higher overall DISCERN scores relative to those from outside the United States (1.21 vs 1.04, *P* < .001). Raw scores by video and mean/median scores by question are included in Supplemental Files A and B, available online, respectively.

### Factors Associated With Overall Video Quality (DISCERN Score)

A univariate linear regression of factors associated with increased overall DISCERN score is delineated in Table 2. Factors associated with significantly higher DISCERN score included number of account subscribers (*β* = 0.02, 95% CI = [0.003‐0.03], *P* = .019), videos posted to health care group accounts (*β* = 0.69, 95% CI = [0.16‐1.22], *P* = .011), videos about patient experience with laryngectomy (*β* = 0.85, 95% CI = [0.09‐1.60], *P* = .029), videos featuring physicians (*β* = 1.42, 95% CI = [0.83‐2.00], *P* < .001), and videos originating from the United Staes (*β* = 1.06, 95% CI = [0.53‐1.59], *P* < .001). However, videos posted on physician accounts, which do not all explicitly feature physicians, had significantly lower odds of a higher DISCERN score compared to other account types (*β* = −0.99, 95% CI = [−1.49 to −0.49], *P* < .001), as did operative videos (*β* = −1.70, 95% CI = [−2.08 to −1.32], *P* < .001). The remainder of the variables showed no significant association with overall DISCERN score, as shown in Table 2.

**Table 2:** Univariate Linear Regression of Factors Associated With Increased Overall DISCERN Score

Characteristic	*β*	95% CI	*P* value
Video and account metrics			
Length (per 10 min)	0.06	[−0.06 to 0.191]	.327
Age of video (per year)	0.01	[−0.05 to 0.08]	.723
Likes (per 100)	0.03	[−0.14 to 0.20]	.708
Views (per 10,000)	0.06	[−0.03 to 0.15]	.216
Comments (per 10)	−0.05	[−0.20 to 0.09]	.466
Account subscribers (per 10,000)	0.02	[0.003 to 0.03]	.019
Account age (per year)	0.02	[−0.04‐0.08]	.580
Type of account			
Physician	−0.99	[−1.49 to −0.49]	<.001
Health care group	0.69	[0.16‐1.22]	.011
Academic institution	−1.03	[−2.72 to 0.67]	.233
Device company	−1.01	[−3.41 to 1.39]	.403
Academic society	1.10	[0.18‐2.01]	.019
Entertainment	1.02	[−0.67 to 2.73]	.233
Video type			
Patient experience	0.85	[0.09‐1.60]	.029
Education from patient	0.56	[−0.32 to 1.44]	.214
Education from physician	1.42	[0.83‐2.00]	<.001
Operation	−1.70	[−2.08 to −1.32]	<.001
Entertainment	1.54	[−0.14‐3.22]	.072
United States	1.06	[0.53‐1.59]	<.001
Otolaryngologist featured	−0.74	[−1.50 to 0.02]	.056

Abbreviation: CI, confidence interval.

Our multivariable linear regression (Table 3) revealed 2 characteristics significantly and independently associated with overall DISCERN score. The number of account subscribers (*β* = 0.02, 95% CI = [0.01‐0.03], *P* = .005) were associated with a higher DISCERN score, while operative videos were associated with a lower DISCERN score (*β* = −1.63, 95% CI = [−2.03 to −1.24], *P* < .001).

**Table 3:** Multivariable Linear Regression of Factors Associated With Increased Overall DISCERN Score

Characteristic	*β*	95% CI	*P* value
Account subscribers (per 10,000)	0.02	[0.01‐0.03]	.005
Operative video	−1.63	[−2.03 to −1.24]	<.001
United States	0.15	[−0.30 to 0.60]	.512

## Discussion

This study evaluated the quality of laryngectomy‐related YouTube videos with the use of the DISCERN grading scale and hypothesized content from health care organizations and physicians would rate higher relative to content from other sources. Our 78 videos were primarily from outside the United States (68.95%) and frequently featured otolaryngologists (85.90%). On average, these videos demonstrated low scores across all 3 subsections outlined by the DISCERN criteria: reliability, quality of treatment information, and overall video quality (mean = 1.97, SD = 1.12). Whereas operative videos (*P* < .001) and those posted to physician‐owned accounts (*P* < .001) were associated with significantly lower DISCERN scores, videos from the United States (*P* < .001), and physician‐featuring videos (*P* < .001) were associated with significantly higher DISCERN scores. In a multivariable linear regression, number of account subscribers (*P* < .001) and videos originating from the United States (*P* = .004) were associated with higher DISCERN score, while operative videos were associated with lower DISCERN score (*P* < .001).

The ubiquity of social media in modern society cannot be understated. A 2021 report by the Pew Research Center found social media use has drastically increased from 50% of Americans in 2011 to 72% in 2021, a value that will only continue to increase.^20^ YouTube was the most frequently visited website, with 83% of Americans reporting ever using it and 46% reporting visiting at least weekly. These values dwarf second‐place Facebook's 69% use rate and Instagram's 40% use rate. YouTube is also highly popular among older US adults and serves as the most popular platform among 50‐ to 64‐year‐old Americans (83%) and only marginally behind Facebook (50% vs 49%) among adults 65 years and older.

Social media's pervasive nature has undoubtedly spread into health care, with a vast majority of American adults reporting seeking health care information online and up to 25% learning from others' medical experiences online.^21^, ^22^ Social media has been discussed as a promising tool for patient education in other surgical fields such as neurosurgery,^23^ and has been used by otolaryngology patients seeking medical advice and searching for appropriate care.^24^, ^25^

Although social media's high prevalence and use rate allow for rapid and widespread dissemination of medical information, its utility is currently limited by a lack of oversight to ensure content reliability and accuracy. This concern held true in our study as we found YouTube videos related to laryngectomy were found to be of moderately low reliability and quality. This result may be surprising, as most videos were posted to accounts owned by either physicians (43.59%) or health care groups (41.03%), and featured otolaryngologists (85.90%). However, these findings are consistent with previous studies which have found comparably low DISCERN scores when evaluating content on YouTube about other otolaryngologic topics such as cholesteatoma,^26^ hypoglossal nerve stimulation,^27^ trigeminal neuralgia,^28^ and functional endoscopic sinus surgery.^29^ Low quality and reliability of content on YouTube has been reported in other specialties as well,^30^, ^31^ although videos about osteoporosis and keratoconus have been found to be of higher quality.^32^, ^33^ Thus, while it is certainly possible for health‐related videos to be of high quality, video topics meeting this requirement are the exception, not the norm. As physicians possess the clinical expertise to explain these topics as trusted sources, they must continue to educate others outside of clinic appointments and bedside visits. Whereas current physician‐led efforts to increase public awareness of various medical conditions are certainly admirable, our findings suggest there may be a benefit to physicians adjusting how they communicate with the general public. An additive effect may be achieved by increasing the quantity of high‐quality content published by physicians on social media platforms like YouTube, as measured by a validated educational grading system. While these efforts certainly are a less traditional component of clinical training, they are still merited given the ubiquity and utility of social media use in patient health information‐seeking behaviors.

We found laryngectomy video overall DISCERN scores vary by video type. Operative videos were found to be of significantly lower quality relative to their nonoperative counterparts—a relationship that held true in our multivariable linear regression. This finding is consistent with the plastic surgery literature,^34^ and is likely due to operative videos' nonpatient target audience. These videos are most effective at showcasing surgical anatomy and technique to otolaryngology trainees and attending physicians, rather than helping patients learn about laryngectomies. As such, they do not meet many aspects of DISCERN criteria because these objectives do not contribute to the operative video's primary goal of educating surgeons. However, it is important to include these videos in our assessment of overall laryngectomy YouTube content, as the videos are easily accessible to patients and will be viewed by them as they seek information about the procedure.

Videos posted on physician YouTube accounts were of significantly lower quality and reliability relative to those posted on nonphysician accounts; however, videos featuring physicians, particularly those from the United States, achieved a significantly higher DISCERN score relative to their nonphysician and international counterparts. While an initially confusing finding, this phenomenon is likely due to the difference between videos physicians post on their personal accounts, which may not always feature themselves, and videos physicians are featured in, which may not always be posted to physician accounts. Whereas many physicians postoperative videos to their personal YouTube channels, videos featuring physicians are often more focused on delivering educational content.

While our cohort of videos represents all laryngectomy videos found on YouTube using a comprehensive list of search terms at the time of data collection, our sample size of 78 is still rather small. This is understandable given the relative rarity of laryngeal cancer relative to other forms of cancer and medical pathologies. Nevertheless, the paucity of videos available is limiting for patients hoping to learn more about the nature of their condition online and is further exacerbated when focusing on nonoperative videos from the United States. Nonetheless, our findings can help physicians recommend the highest quality of laryngectomy educational videos available to patients and help clinicians understand the quality of laryngectomy‐related content patients are discovering on their own.

However, we also uncover an acute need to produce additional, higher quality videos about laryngectomy to expand the limited pool of supplemental and accessible educational resources patients have available to them. YouTube itself has taken efforts to direct its audience to accurate sources of health information by verifying trusted and accurate health‐focused YouTube channels as health‐related authorities.^35^, ^36^ Similarly, the American Medical Association has called for efforts to stymie medical misinformation on social media in light of public response to the COVID‐19 pandemic.^37^ While these efforts may increase the quality of health‐related information and sources available online, health care institutions and otolaryngologists may contribute to this cause by publishing high‐quality content about laryngectomy to highly trafficked social media platforms to ensure high‐quality content remains readily accessible to patients online.

The main limitation of this study was the use of a nonotolaryngology‐specific grading system applied to a single, albeit the largest and most‐frequented, online video content platform. One recent study highlighted a novel evaluation model derived from the IVORY Guidelines titled, “Instructional Videos in Otorhinolaryngology by YO‐IFOS (IVORY)‐grading‐system (GS),” which pose established consensus recommendations to produce educational surgical videos in otolaryngology.^38^ However, IVORY‐GS remains better suited for physician‐focused educational content, whereas DISCERN is intended for assessing patient‐focused educational content. Additionally, this study solely evaluated video content and did not explore the ability of each video to educate its audience. It is important to consider the videos evaluated in our study were not all created with the aim of educating patients. Videos created for this purpose may be exclusively found on dedicated academic institution‐specific websites, which were not evaluated here. Future studies applying the IVORY‐GS may prove a superior method when it comes to producing and evaluating educational video content in otolaryngology. Moreover, future work should consider how video content translates to meaningful patient education, effects on surgical outcomes, and shared‐treatment decision‐making. Interestingly, videos created by patients and those describing the patient experience after laryngectomy earned higher DISCERN scores. These findings advocate for strong patient input when deciding how to compose future educational videos. Video content is a wonderful supplement to the patient experience, but it is no substitute for active patient involvement in their personal care. Surveying patients in waiting rooms or clinics may guide future education goals, optimize delivery methods, and identify gaps in patient health literacy. Advocating for accessible and transparent lines of communication may ultimately bridge any potential void in the knowledge otolaryngologists share with their patients and what patients want to know.

## Conclusion

Using the DISCERN tool, we found the quality and quantity of YouTube content related to laryngectomy is poor. Despite high levels of accessibility and interaction with content on YouTube, there is an acute need for comprehensive and reliable educational videos.

## Author Contributions

**Matthew E. Lin**, conception and design of work, data acquisition and analysis, interpretation of data, drafting of manuscript, critical revision; **Oluwatobiloba Ayo‐Ajibola**, data acquisition and analysis, interpretation of data, drafting of manuscript, critical revision; **Carlos X. Castellanos**, data acquisition and analysis, interpretation of data, drafting of manuscript, critical revision; **Jonathan West**, interpretation of data, drafting of manuscript, critical revision; **Neil Luu**, interpretation of data, drafting of manuscript, critical revision; **Ian Kim**, interpretation of data, critical revision; **Niels C. Kokot**, conception and design of work, interpretation of data, critical revision.

## Disclosures

### Competing interests

None.

### Funding source

None.

## Supporting information


**Supplement A**. Raw video DISCERN scores by individual question and overall score (Question 16).

**Supplement B**. Summary Statistics of all videos analyzed stratified by individual DISCERN criteria.
