# Supplementary material for: Assessing Laryngectomy Patient Education on YouTube: Investigating Quality and Reliability
Source: OTO Open. 2024 Jan 31;8(1):e113. doi: 10.1002/oto2.113 (PMC10828916; doi:10.1002/oto2.113)
Supplement: Supplementary file 1 — Supplement A. Raw video DISCERN scores by individual question and overall score (Question 16). [file OTO2-8-e113-s002.docx]

| Youtube Link | Discern 1 | Discern 2 | Discern 3 | Discern 4 | Discern 5 | Discern 6 | Discern 7 | Discern 8 | Discern 9 | Discern 10 | Discern 11 | Discern 12 | Discern 13 | Discern 14 | Discern 15 | Discern 16 |
| --- | --- | --- | --- | --- | --- | --- | --- | --- | --- | --- | --- | --- | --- | --- | --- | --- |
| https://www.youtube.com/watch?v=LYrIVn4elQY | 4 | 4 | 5 | 1 | 1 | 4 | 2 | 3 | 4 | 3 | 3 | 2 | 4 | 4 | 3 | 3 |
| https://www.youtube.com/watch?v=xmRHUXD3URk | 4 | 5 | 5 | 2 | 2 | 3 | 2 | 3 | 4 | 2 | 1 | 2 | 2 | 5 | 3 | 3 |
| https://www.youtube.com/watch?v=lwxIBk0WSYY | 5 | 5 | 5 | 2 | 2 | 2 | 3 | 2 | 4 | 2 | 1 | 1 | 2 | 5 | 1 | 3 |
| https://www.youtube.com/watch?v=5t5J48NuImY | 5 | 3 | 3 | 1 | 2 | 3 | 2 | 3 | 5 | 5 | 3 | 1 | 5 | 4 | 2 | 3 |
| https://www.youtube.com/watch?v=d3W4TaeUsTA | 4 | 3 | 4 | 2 | 2 | 1 | 2 | 2 | 1 | 2 | 1 | 2 | 2 | 1 | 1 | 1 |
| https://www.youtube.com/watch?v=CXuxQQI_9SU | 5 | 4 | 2 | 1 | 2 | 2 | 2 | 1 | 4 | 1 | 1 | 1 | 1 | 1 | 1 | 1 |
| https://www.youtube.com/watch?v=Lruj-mjIn8I | 5 | 4 | 3 | 2 | 2 | 2 | 2 | 1 | 1 | 4 | 1 | 4 | 2 | 1 | 1 | 1 |
| https://www.youtube.com/watch?v=gwQ7MPjdmoo | 5 | 4 | 4 | 2 | 2 | 4 | 2 | 3 | 3 | 3 | 2 | 2 | 3 | 4 | 2 | 3 |
| https://www.youtube.com/watch?v=H9sro0vfd8I | 5 | 5 | 4 | 2 | 2 | 2 | 1 | 3 | 5 | 5 | 2 | 1 | 4 | 5 | 1 | 4 |
| https://www.youtube.com/watch?v=5haB5_abbMk | 5 | 5 | 4 | 1 | 2 | 1 | 2 | 4 | 5 | 1 | 4 | 1 | 1 | 2 | 1 | 2 |
| https://www.youtube.com/watch?v=u6tma0igkD0 | 5 | 5 | 4 | 1 | 2 | 2 | 2 | 1 | 4 | 1 | 1 | 1 | 1 | 1 | 1 | 1 |
| https://www.youtube.com/watch?v=WQfXc4FprfA | 5 | 4 | 5 | 2 | 2 | 4 | 2 | 3 | 4 | 3 | 3 | 3 | 2 | 2 | 1 | 3 |
| https://www.youtube.com/watch?v=AiO-3N8zZrE | 5 | 4 | 5 | 1 | 2 | 4 | 1 | 3 | 4 | 3 | 2 | 2 | 1 | 2 | 1 | 2 |
| https://www.youtube.com/watch?v=W4m7QbKiES4 | 4 | 2 | 4 | 1 | 1 | 4 | 2 | 4 | 3 | 2 | 3 | 1 | 1 | 2 | 1 | 1 |
| https://www.youtube.com/watch?v=qVBRINp4cfQ | 5 | 4 | 5 | 1 | 2 | 3 | 1 | 2 | 4 | 3 | 2 | 2 | 1 | 2 | 1 | 2 |
| https://www.youtube.com/watch?v=ntcEDCP1UaA | 3 | 3 | 4 | 1 | 1 | 2 | 1 | 2 | 2 | 1 | 1 | 1 | 2 | 2 | 2 | 1 |
| https://www.youtube.com/watch?v=n_nwHvrvQs0 | 3 | 2 | 4 | 2 | 1 | 3 | 2 | 3 | 2 | 1 | 1 | 1 | 3 | 2 | 2 | 2 |
| https://www.youtube.com/watch?v=zZO7SN3J6_w | 4 | 4 | 4 | 3 | 2 | 4 | 3 | 3 | 3 | 3 | 3 | 1 | 4 | 2 | 2 | 3 |
| https://www.youtube.com/watch?v=oqj4f396Aaw | 4 | 4 | 5 | 2 | 1 | 4 | 1 | 3 | 4 | 3 | 4 | 2 | 4 | 4 | 2 | 3 |
| https://www.youtube.com/watch?v=G3PkgWGR4lQ | 2 | 2 | 1 | 1 | 1 | 1 | 1 | 2 | 1 | 1 | 2 | 1 | 2 | 2 | 1 | 1 |
| https://www.youtube.com/watch?v=AvEe472khR4 | 3 | 3 | 1 | 1 | 1 | 1 | 1 | 2 | 1 | 1 | 2 | 1 | 2 | 1 | 1 | 1 |
| https://www.youtube.com/watch?v=XpLSWB2rUNU | 5 | 4 | 5 | 4 | 2 | 4 | 3 | 2 | 3 | 2 | 1 | 1 | 1 | 3 | 2 | 2 |
| https://www.youtube.com/watch?v=OS6KR5Wlzgo | 5 | 5 | 5 | 2 | 2 | 2 | 2 | 1 | 5 | 3 | 3 | 1 | 3 | 5 | 1 | 3 |
| https://www.youtube.com/watch?v=h2zminCImyw | 5 | 5 | 5 | 2 | 2 | 1 | 2 | 5 | 5 | 5 | 3 | 1 | 5 | 5 | 3 | 5 |
| https://www.youtube.com/watch?v=oKUvMsUK_AI | 5 | 5 | 4 | 2 | 2 | 1 | 2 | 5 | 3 | 4 | 5 | 5 | 5 | 5 | 1 | 5 |
| https://www.youtube.com/watch?v=9GeNF00PkEg | 4 | 5 | 4 | 3 | 2 | 4 | 3 | 4 | 3 | 4 | 5 | 2 | 3 | 3 | 2 | 4 |
| https://www.youtube.com/watch?v=NvW-KaEj_AU | 3 | 5 | 4 | 1 | 2 | 1 | 1 | 1 | 5 | 3 | 1 | 1 | 1 | 1 | 1 | 1 |
| https://www.youtube.com/watch?v=gzkPaCxyZ5M | 5 | 5 | 5 | 5 | 3 | 2 | 2 | 4 | 5 | 4 | 3 | 1 | 4 | 3 | 1 | 4 |
| https://www.youtube.com/watch?v=rCWea0cV_28 | 4 | 4 | 4 | 2 | 1 | 4 | 1 | 3 | 4 | 3 | 3 | 2 | 2 | 2 | 1 | 3 |
| https://www.youtube.com/watch?v=YN1gdWPh1M0&t=924s | 4 | 4 | 4 | 4 | 4 | 5 | 3 | 4 | 4 | 4 | 4 | 3 | 2 | 3 | 2 | 4 |
| https://www.youtube.com/watch?v=_OkjLkh98tQ | 2 | 3 | 5 | 2 | 2 | 3 | 2 | 4 | 3 | 3 | 3 | 4 | 4 | 4 | 4 | 4 |
| https://www.youtube.com/watch?v=9vo_kaGzfEY | 4 | 5 | 2 | 2 | 2 | 1 | 1 | 1 | 2 | 1 | 1 | 1 | 1 | 1 | 1 | 1 |
| https://www.youtube.com/watch?v=ss-yVoK9Oso | 3 | 4 | 4 | 1 | 1 | 3 | 1 | 3 | 3 | 3 | 1 | 1 | 3 | 4 | 2 | 3 |
| https://www.youtube.com/watch?v=hS77FmF5hr4 | 2 | 2 | 2 | 1 | 1 | 2 | 1 | 1 | 1 | 1 | 1 | 1 | 1 | 1 | 1 | 1 |
| https://www.youtube.com/watch?v=6q9q4umrBAI | 3 | 3 | 3 | 1 | 1 | 3 | 1 | 1 | 3 | 1 | 1 | 1 | 2 | 3 | 2 | 2 |
| https://www.youtube.com/watch?v=ksfFN9ZwfPA | 3 | 3 | 3 | 1 | 1 | 3 | 1 | 1 | 2 | 3 | 1 | 1 | 3 | 2 | 2 | 2 |
| https://www.youtube.com/watch?v=LCVhjiba8hs | 2 | 2 | 3 | 1 | 1 | 3 | 1 | 2 | 2 | 3 | 4 | 1 | 2 | 4 | 2 | 2 |
| https://www.youtube.com/watch?v=k31RJg95kCE | 2 | 2 | 2 | 1 | 1 | 2 | 1 | 1 | 1 | 1 | 1 | 1 | 1 | 1 | 1 | 1 |
| https://www.youtube.com/watch?v=s95hPv5fFYU | 3 | 3 | 5 | 1 | 1 | 3 | 1 | 1 | 3 | 4 | 1 | 1 | 3 | 1 | 3 | 2 |
| https://www.youtube.com/watch?v=C7MjzqUu4TA | 4 | 4 | 3 | 2 | 2 | 4 | 2 | 4 | 3 | 3 | 4 | 2 | 4 | 5 | 2 | 3 |
| https://www.youtube.com/watch?v=LbbTy0CPzx0 | 4 | 4 | 2 | 3 | 4 | 4 | 3 | 3 | 4 | 2 | 3 | 3 | 2 | 3 | 1 | 3 |
| https://www.youtube.com/watch?v=AVKZ3NEMBl4 | 2 | 2 | 1 | 1 | 1 | 1 | 1 | 1 | 1 | 2 | 1 | 1 | 1 | 1 | 1 | 1 |
| https://www.youtube.com/watch?v=VFpsHLY6QAo | 2 | 2 | 1 | 1 | 1 | 1 | 1 | 1 | 1 | 2 | 1 | 1 | 1 | 1 | 1 | 1 |
| https://www.youtube.com/watch?v=XFDM_LoDyLM | 5 | 4 | 3 | 4 | 4 | 3 | 3 | 4 | 3 | 2 | 2 | 2 | 1 | 4 | 2 | 3 |
| https://www.youtube.com/watch?v=ZXTZHi_X-NU | 2 | 2 | 2 | 1 | 1 | 1 | 1 | 1 | 1 | 2 | 1 | 2 | 2 | 2 | 1 | 1 |
| https://www.youtube.com/watch?v=a-pZzLUzo9I | 4 | 5 | 4 | 3 | 3 | 4 | 1 | 3 | 4 | 4 | 3 | 2 | 3 | 4 | 3 | 4 |
| https://www.youtube.com/watch?v=AMRlGzkLTUI | 4 | 4 | 3 | 5 | 5 | 4 | 2 | 4 | 2 | 3 | 4 | 2 | 3 | 4 | 4 | 4 |
| https://www.youtube.com/watch?v=rg0EG_cPA8E | 3 | 4 | 2 | 1 | 2 | 3 | 1 | 4 | 2 | 2 | 2 | 3 | 3 | 4 | 3 | 3 |
| https://www.youtube.com/watch?v=CNg9IArtuH0 | 2 | 4 | 2 | 1 | 1 | 1 | 1 | 2 | 1 | 2 | 1 | 2 | 1 | 2 | 1 | 1 |
| https://www.youtube.com/watch?v=Vm91dPvyaZQ | 2 | 2 | 3 | 1 | 1 | 3 | 1 | 1 | 2 | 1 | 1 | 1 | 1 | 1 | 1 | 1 |
| https://www.youtube.com/watch?v=mE_Xz2Bax_c | 1 | 1 | 3 | 1 | 1 | 3 | 1 | 1 | 1 | 1 | 1 | 1 | 1 | 1 | 1 | 1 |
| https://www.youtube.com/watch?v=6tHYLzKV1rs | 4 | 5 | 2 | 4 | 2 | 2 | 2 | 1 | 1 | 1 | 1 | 1 | 1 | 1 | 1 | 1 |
| https://www.youtube.com/watch?v=aUdOdsBf4zM | 5 | 4 | 3 | 3 | 4 | 4 | 5 | 4 | 4 | 4 | 3 | 4 | 4 | 4 | 3 | 4 |
| https://www.youtube.com/watch?v=HlM8SGnZ7Z8 | 4 | 4 | 3 | 1 | 2 | 1 | 2 | 1 | 1 | 1 | 1 | 1 | 1 | 4 | 1 | 1 |
| https://www.youtube.com/watch?v=EIzsWoWMs4I | 2 | 3 | 2 | 1 | 1 | 1 | 1 | 1 | 2 | 1 | 1 | 1 | 1 | 1 | 1 | 1 |
| https://www.youtube.com/watch?v=Z80mbOijjaI | 3 | 3 | 2 | 1 | 2 | 1 | 1 | 2 | 2 | 1 | 2 | 1 | 1 | 1 | 1 | 1 |
| https://www.youtube.com/watch?v=xQ9E44uyrKw | 2 | 3 | 2 | 1 | 2 | 1 | 1 | 1 | 1 | 2 | 1 | 1 | 1 | 2 | 1 | 1 |
| https://www.youtube.com/watch?v=uU8bO4QJUG8 | 3 | 4 | 1 | 1 | 2 | 1 | 1 | 2 | 2 | 1 | 2 | 1 | 1 | 1 | 2 | 1 |
| https://www.youtube.com/watch?v=eWVMV0wpR4M | 2 | 2 | 1 | 1 | 1 | 1 | 1 | 2 | 1 | 1 | 2 | 1 | 2 | 1 | 1 | 1 |
| https://www.youtube.com/watch?v=zGjYMO_wWRE | 4 | 5 | 3 | 2 | 2 | 3 | 2 | 3 | 4 | 3 | 3 | 2 | 2 | 4 | 2 | 3 |
| https://www.youtube.com/watch?v=ww85ao4fElQ | 2 | 3 | 2 | 1 | 2 | 1 | 1 | 2 | 3 | 2 | 1 | 1 | 1 | 1 | 1 | 1 |
| https://www.youtube.com/watch?v=unj8IuMV2-4 | 3 | 3 | 1 | 1 | 1 | 1 | 1 | 2 | 1 | 1 | 2 | 1 | 2 | 1 | 1 | 1 |
| https://www.youtube.com/watch?v=lX6hyP5z6Ds | 4 | 4 | 3 | 1 | 2 | 1 | 1 | 1 | 2 | 1 | 2 | 1 | 2 | 1 | 1 | 1 |
| https://www.youtube.com/watch?v=2ZKOv4w0_6k | 2 | 2 | 2 | 1 | 1 | 1 | 1 | 1 | 1 | 1 | 1 | 1 | 1 | 1 | 1 | 1 |
| https://www.youtube.com/watch?v=qi5Dhhz_8v4 | 2 | 2 | 2 | 1 | 2 | 1 | 1 | 1 | 1 | 1 | 1 | 1 | 1 | 1 | 1 | 1 |
| https://www.youtube.com/watch?v=TW2FCKV1EXE | 2 | 2 | 2 | 1 | 2 | 1 | 1 | 1 | 1 | 1 | 1 | 1 | 1 | 1 | 1 | 1 |
| https://www.youtube.com/watch?v=Jhx9TzFCByo | 2 | 2 | 2 | 1 | 2 | 1 | 1 | 1 | 1 | 1 | 1 | 1 | 1 | 1 | 1 | 1 |
| https://www.youtube.com/watch?v=hS77FmF5hr4 | 2 | 2 | 2 | 1 | 2 | 1 | 1 | 1 | 1 | 1 | 1 | 1 | 1 | 1 | 1 | 1 |
| https://www.youtube.com/watch?v=j_jj6yz8DZ0 | 3 | 3 | 2 | 1 | 2 | 3 | 2 | 2 | 2 | 1 | 1 | 1 | 1 | 1 | 1 | 2 |
| https://www.youtube.com/watch?v=nO-n7dRbmY8 | 4 | 4 | 4 | 2 | 2 | 4 | 2 | 3 | 4 | 4 | 3 | 2 | 2 | 1 | 5 | 4 |
| https://www.youtube.com/watch?v=ajlXBgDlTK0 | 3 | 3 | 2 | 1 | 1 | 1 | 1 | 1 | 1 | 2 | 1 | 1 | 1 | 1 | 1 | 1 |
| https://www.youtube.com/watch?v=qhDcbh1s3UY | 4 | 4 | 3 | 1 | 2 | 2 | 1 | 3 | 1 | 2 | 2 | 1 | 1 | 2 | 1 | 2 |
| https://www.youtube.com/watch?v=3pQDadjO-As | 2 | 2 | 2 | 1 | 2 | 1 | 1 | 1 | 1 | 1 | 1 | 1 | 1 | 1 | 1 | 1 |
| https://www.youtube.com/watch?v=ExskSIfNrFY | 3 | 3 | 2 | 1 | 2 | 1 | 2 | 1 | 2 | 1 | 1 | 1 | 1 | 1 | 1 | 1 |
| https://www.youtube.com/watch?v=esaoM3ZXw70 | 3 | 3 | 2 | 1 | 2 | 1 | 1 | 1 | 2 | 1 | 1 | 1 | 2 | 2 | 1 | 1 |
| https://www.youtube.com/watch?v=lCxFj5rdw2s | 2 | 2 | 2 | 1 | 1 | 1 | 1 | 1 | 1 | 2 | 1 | 1 | 1 | 1 | 1 | 1 |
| https://www.youtube.com/watch?v=wpQKuZyxduY | 3 | 2 | 2 | 1 | 2 | 1 | 1 | 1 | 2 | 1 | 1 | 1 | 1 | 1 | 1 | 1 |
| https://www.youtube.com/watch?v=uxfjjEmtDFA | 3 | 3 | 2 | 1 | 1 | 1 | 1 | 1 | 1 | 2 | 1 | 1 | 2 | 2 | 1 | 1 |
