# Supplementary material for: Assessing Laryngectomy Patient Education on YouTube: Investigating Quality and Reliability
Source: OTO Open. 2024 Jan 31;8(1):e113. doi: 10.1002/oto2.113 (PMC10828916; doi:10.1002/oto2.113)
Supplement: Supplementary file 2 — Supplement B. Summary Statistics of all videos analyzed stratified by individual DISCERN criteria. [file OTO2-8-e113-s001.docx]

| **Discern Criteria (1-5 scale)** | **Mean (SD)** | **SD** | **Median** |
| --- | --- | --- | --- |
| 1. Are the aims clear? | 3.42 | 1.16 | 3 |
| 2. Does it achieve its aims? | 3.44 | 1.10 | 4 |
| 3. Is it relevant? | 3.04 | 1.26 | 3 |
| 4. Is it clear what sources of information... | 1.62 | 1.00 | 1 |
| 5. Is it clear when the information used... | 1.81 | 0.82 | 2 |
| 6. Is it balanced and unbiased? | 2.23 | 1.24 | 2 |
| 7. Does it provide details of additional... | 1.55 | 0.77 | 1 |
| 8. Does it refer to areas of uncertainty? | 2.15 | 1.20 | 2 |
| 9. Does it describe how each treatment works? | 2.51 | 1.37 | 2 |
| 10. Does it describe the benefits of each treatment? | 2.17 | 1.19 | 2 |
| 11. Does it describe the risks of each treatment? | 1.90 | 1.12 | 1 |
| 12. Does it describe what would happen if no treatment is used? | 1.49 | 0.86 | 1 |
| 13. Does it describe how the treatment choices affect overall quality of life? | 1.99 | 1.17 | 2 |
| 14. Is it clear that there may be more than one possible treatment choice? | 2.27 | 1.41 | 2 |
| 15. Does it provide support for shared decision-making? | 1.53 | 0.86 | 2 |
